# *Cis*-acting super-enhancer lncRNAs as biomarkers to early-stage breast cancer

**DOI:** 10.1186/s13058-021-01479-8

**Published:** 2021-10-30

**Authors:** Ali S. Ropri, Rebecca S. DeVaux, Jonah Eng, Sridar V. Chittur, Jason I. Herschkowitz

**Affiliations:** 1 Department of Biomedical Sciences, Cancer Research Center, University at Albany, 1 Discovery Drive, Suite 317, Rensselaer, NY 12144 USA; 2 Bethlehem Central High School, Bethlehem Central School District, Delmar, NY 12054 USA; 3 Center for Functional Genomics, Cancer Research Center, University at Albany, Rensselaer, NY 12144 USA

**Keywords:** Breast cancer progression, Ductal carcinoma in situ, Super-enhancer long non-coding RNAs, Super-enhancers

## Abstract

**Background:**

Increased breast cancer screening over the past four decades has led to a substantial rise in the diagnosis of ductal carcinoma in situ (DCIS). Although DCIS lesions precede invasive ductal carcinoma (IDC), they do not always transform into cancer. The current standard-of-care for DCIS is an aggressive course of therapy to prevent invasive and metastatic disease resulting in over-diagnosis and over-treatment. Thus, there is a critical need to identify functional determinants of progression of DCIS to IDC to allow discrimination between indolent and aggressive disease. Recent studies show that super-enhancers, in addition to promoting other gene transcription, are themselves transcribed producing super-enhancer associated long noncoding RNAs (SE-lncRNAs). These SE-lncRNAs can interact with their associated enhancer regions in *cis* and influence activities and expression of neighboring genes. Furthermore, they represent a novel, untapped group of therapeutic targets.

**Methods:**

With an integrative analysis of enhancer loci with global expression of SE-lncRNAs in the MCF10A progression series, we have identified differentially expressed SE-lncRNAs which can identify mechanisms for DCIS to IDC progression. Furthermore, cross-referencing these SE-lncRNAs with patient samples in the The Cancer Genome Atlas (TCGA) database, we have unveiled 27 clinically relevant SE-lncRNAs that potentially interact with their enhancer to regulate nearby gene expression. To complement SE-lncRNA expression studies, we conducted an unbiased global analysis of super-enhancers that are acquired or lost in progression.

**Results:**

Here we designate SE-lncRNAs RP11-379F4.4 and RP11-465B22.8 as potential markers of progression of DCIS to IDC through regulation of the expression of their neighboring genes (RARRES1 and miR-200b, respectively). Moreover, we classified 403 super-enhancer regions in MCF10A normal cells, 627 in AT1, 1053 in DCIS, and 320 in CA1 cells. Comparison analysis of acquired/lost super-enhancer regions with super-enhancer regions classified in 47 ER positive patients, 10 triple negative breast cancer (TNBC) patients, and 11 TNBC cell lines reveal critically acquired pathways including STAT signaling and NF-kB signaling. In contrast, protein folding, and local estrogen production are identified as major pathways lost in progression.

**Conclusion:**

Collectively, these analyses identify differentially expressed SE-lncRNAs and acquired/lost super-enhancers in progression of breast cancer important for promoting DCIS lesions to IDC.

**Supplementary Information:**

The online version contains supplementary material available at 10.1186/s13058-021-01479-8.

## Background

Breast cancer can be defined as a group of diseases with heterogeneous origins, molecular profiles and behaviors characterized by uncontrolled proliferation of cells within the mammary gland. Around one in eight women in the USA will develop breast cancer in their lifetime, making it the second most frequently diagnosed cancer behind skin cancer [1]. In 2021, an estimated 281,550 cases of invasive breast carcinoma are predicted to be diagnosed, and over 40,000 deaths are expected, accounting for almost 7% of all cancer mortality each year. Ductal carcinoma in situ (DCIS) is the presence of abnormal cells inside a milk duct in the breast and is a precursor to invasive cancer. DCIS accounts for 20% of breast cancer diagnoses per year [2], however, while not all DCIS lesions progress to invasive cancer, all are treated as such leading to overdiagnosis and overtreatment. In fact, DCIS lesions sometimes grow so slowly that even without treatment it would not affect a woman’s health. Long-term studies have found that only 40% of women with untreated DCIS are ultimately diagnosed with invasive breast cancer [3]. The steep increase in diagnosis of DCIS over the past 30–40 years is believed to be a result of more frequent mammography [4]. However, because over half of these in situ lesions will not progress to invasive breast cancer, controversies have arisen about approaches to treatment.

As early screening is advocated and on the rise, better understanding of the progression of non-invasive to invasive breast cancer is a prerequisite for correct diagnosis of patients. There is a need to highlight functional determinants DCIS progression to invasive ductal carcinoma (IDC) thus allowing discrimination between indolent and potentially metastatic breast cancers. Understanding the mechanisms of transition of normal breast to invasive breast cancer can have significant implications for preventive and clinical management of breast cancer.

Transcriptome reprogramming is one of the crucial characteristics of cancer, where aberrant gene expression promotes tumor initiation, progression, and metastasis. This can be amplified by *cis*-element changes in noncoding genomic regions [5]. For example, super-enhancers (SEs), also known as stretch enhancers, are genomic regions where multiple enhancers are clustered together. They exert more potent effects than typical enhancers, are characterized by high levels of Mediator binding, and are associated primarily with tissue-specific genes [6]. Super-enhancers are most likely the major contributors to the expression of their associated genes [7]. There have also been several indications of links between super-enhancers and diseases [8]. Recent studies have shown that SEs play key roles in determining cell identity in both healthy and pathological states. Over 25,000 enhancers were identified as differentially activated in renal, breast, and prostate tumor cells, as compared with normal cells. This suggests a network between malignancy and enhancer activity [9]. Likewise, cancer cells have been shown to acquire super-enhancers at oncogenes and cancerous phenotype relies on the abnormal transcription propelled by Ses [10]. Additionally, super-enhancer regions are transcribed generating long non-coding RNAs (lncRNAs) that may play a pivotal role in assisting the super-enhancer function [11].

Long non-coding RNAs are a large class of non-coding transcripts that are > 200 nucleotides in length and do not encode proteins [12]. Evidence has indicated that lncRNAs regulate gene expression at the levels of epigenetic modification, transcription, translation, and post-translation [13]. At the transcription level, these RNAs can be associated with super-enhancer regions, and interact with enhancer sequences to influence activities of neighboring genes. A study published in 2011 shows that enhancer associated lncRNA (e-lncRNA) HOTTIP, which resides at the 5′ tip of the HOXA locus, regulates the transcription of various HOXA genes in vivo through chromosomal looping of its enhancer to the promoter region of these genes [14]. In the same fashion, Sigova et al. observe that nascent e-lncRNAs are necessary for the recruitment of Yin-Yang-1 transcription factor to its target enhancer [15]. As a result, e-lncRNAs have been indicated to play a vital role in engaging with transcription factors and localizing them to cognate enhancers. Enhancer lncRNAs are also involved in diverse tumor biological processes, including cell proliferation, apoptosis, invasion, metastasis, and angiogenesis as well as interacting with their enhancers to regulate genes specific to cell identity [16].

Previously, our laboratory has profiled global lncRNA expression in a unique patient‐based model of breast cancer progression, wherein early DCIS lesions are directly contiguous with an IDC lesion. From this unbiased patient‐based model, 132 lncRNAs were identified as differentially expressed in early breast cancer progression [17], of which 78 were transcribed from super-enhancer regions. This statistically significant enrichment in enhancer associated lncRNAs suggests a core mechanism of breast cancer progression. In this study, we identify super-enhancer associated lncRNAs (SE-lncRNAs) that are differentially expressed between non-invasive and invasive breast cancer in the MCF10A progression series as well as DCIS and IDC patient samples. We also designate two of the most promising SE-lncRNAs from our list for their potential *cis*-acting capabilities in regulating nearby gene expression crucial for progression to IDC. Furthermore, we highlight super-enhancers that are acquired or lost in progression to IDC, giving insight about genes and pathways these super-enhancers regulate which may be necessary for progression.

## Materials and methods

### Cell culture

The MCF10A progression series (MCF10A, MCF10A-AT1 (AT1), MCF10A-DCIS (DCIS or DCIS.com), and MCF10A-CA1 (CA1)) were purchased from the Barbara Ann Karmanos Cancer Institute and maintained in a culture of Dulbecco's modified Eagle's medium (DMEM) medium supplemented with 5% horse serum, 20 ng/mL EGF, 0.5 mg/mL hydrocortisone, 100 ng/mL cholera toxin, 10 μg/mL insulin, and 1 × antibiotic‐antimycotic (Gibco, Grand Island, NY). All cell lines were verified by STR analysis and routinely screened for mycoplasma contamination.

### Microarray

Arraystar Super-enhancer lncRNA arrays were used to systematically profile 7753 lncRNAs transcribed from super-enhancer (SE) regions along with 7040 corresponding SE-regulated protein coding genes. Briefly, an optimized mixture of oligo(dT) and random primers, each containing a T7 polymerase promoter, is annealed to the RNA. The cDNA is synthesized by reverse transcription followed by 5’ adapter annealing and PCR amplification. Finally, cyanine 3- or cyanine 5-labeled cRNA is synthesized by in vitro transcription from the T7 promoter by T7 RNA polymerase.

### Gene expression analysis

Gene expression was confirmed for the 27 potentially *cis*-acting SE-lncRNAs and 4 highest differentiated SE-lncRNAs. Forty-eight hours after seeding, total RNA was collected using Omega Bio-Tek E.Z.N.A. Total RNA Kit (Catalog No. R6-834-02). RNA was subsequently reverse-transcribed using Applied Biosystems High-Capacity cDNA Reverse Transcription Kit (Life Technologies Catalog No. 4368813) and analyzed by quantitative reverse transcription PCR (qRT-PCR) with Sybr Green on the QuantStudio 12 K Flex (Thermo Fisher Scientific) for indicated genes. The ΔΔ*C*t method was used to determine gene expression fold change with 18 s, Act-b, and GAPDH used for controls.

### Cellular localization

Whole cell lysate, cytoplasmic fraction, and nuclear fraction were extracted from MCF10A (10A) and MCF10A-CA1 (CA1) cells using Protein and RNA Isolation System (PARIS) Kit (Life technologies Catalog No. AM1921). For whole cell lysate and for each cytoplasmic and nuclear fraction from 10A and CA1 cells, 87.5% of the sample was used for RNA extraction, while 12.5% was used for an immunoblot to ensure the fractionation was done correctly. The 7:1 ratio was used to maximize RNA content as lncRNAs are relatively lowly expressed. GAPDH (Cell Signaling 14C10) was utilized as control for cytoplasmic fraction, while Tri-Methyl Histone H3 (Lys 27) (Cell Signaling C36B11) was used as control for nuclear fraction for the immunoblot. RNA was isolated from each fraction using Omega Bio-Tek E.Z.N.A. Total RNA Kit (Catalog No. R6-834-02). Reverse transcription was performed with the Applied Biosystems High-Capacity cDNA Reverse Transcription Kit (Life Technologies Catalog No. 4368813) on the extracted RNA from each compartment as well as the whole cell lysate and RT-qPCR were carried out to analyze the localization of these SE-lncRNAs within the 10A and CA1 cells as well as fold change. 18 s, Actin-B, HOTAIR were used as controls for localization. 18 s and Actin-B were also used as controls for fold change to validate the change in expression we saw in our microarray data.

### Patient samples extraction

Archived Formalin-Fixed Paraffin-Embedded (FFPE) patient samples were acquired from Reading Hospital, PA. RNA was extracted from 24 DCIS and 24 IDC samples using the miRNeasy FFPE Kit supplied by QIAGEN (Cat. No. 217504). Reverse transcription was performed using Applied Biosystems High-Capacity cDNA Reverse Transcription Kit (Life Technologies Catalog No. 4368813) on the extracted RNA from each patient sample and RT-qPCR was performed to analyze expression of the 14 target SE-lncRNAs. 18 s, Actin-B and GAPDH were used as controls for fold change in each sample. Unpaired t-test was performed to obtain significance between expression of SE-lncRNAs in DCIS versus IDC patient Samples.

### Quantitative reverse transcription PCR primers

TaqMan assays were purchased from Thermo Fisher Scientific (Waltham, MA), 18S (Hs99999901_s1), Actin (Hs99999903_m1), glyceraldehyde 3‐phosphate dehydrogenase (GAPDH; Hs02758991_g1), HOTAIR (Hs03296680), U6 snRNA (Catalog No. 4427975), miR-200b (hsa-miR-200b, Catalog No. 4427975). Sybr‐green assays purchased from Integrated DNA Technologies (Coralville, IA):

**Table:** 

AC053503.6	Forward Primer: 5′-AGGTGGATTAGAGGGGGTGT-3′
	Reverse Primer: 5′-GGCTGAGAAGGGGGTTTCTG-3′
AC068580.7	Forward Primer: 5′-CCCGTCGTGACCTCATTGTG-3′
	Reverse Primer: 5′-GAACCCCTTTTCCTCACCCA-3′
CCND2-AS1	Forward Primer: 5′-CAAGCTGGAACCCTGCAAGA-3′
	Reverse Primer: 5′-AAGGGTATACCTTCCTCCCCA-3′
CTD-2033D15.1	Forward Primer: 5′-GGTAAGAAGCAAAGCCCTGGA3′
	Reverse Primer: 5′-TGGCTGAGACGCCATCTGTA-3′
FAM83H-AS1	Forward Primer: 5′-GCAACACCCTACTGACCTTGT-3′
	Reverse Primer: 5′-AGCTCTGTGGTGACTGTCTT-3′
FAM87A	Forward Primer: 5′-TTCCGCAGGTTTTAGTGGCT-3′
	Reverse Primer: 5′-CAAACTGTCCCCAACTCCCA-3′
GATA2-AS1	Forward Primer: 5′-GACCCTCTGAAAGACACCGC-3′
	Reverse Primer: 5′-TCTTGCTCATGTGTGAGGGC-3′
HCG9	Forward Primer: 5′-CAGGAACCCAGGGACTTCAG-3′
	Reverse Primer: 5′-TGTTCTCTGCAGCTTGACCT-3′
HOXA11-AS	Forward Primer: 5′-TCCGATTTGCACGGTGACTT-3′
	Reverse Primer: 5′-CGGATGTCAGCGCCTCTAAA-3′
LINC00885	Forward Primer: 5′-GGCACTGTAGAAGCCCCATT-3′
	Reverse Primer: 5′-GTCCAGCGAACTGAAGGACA-3′
LINC01125	Forward Primer: 5′-AGGCAAAGATGAGCAGAGCC-3′
	Reverse Primer: 5′-CCAAGCAATGCTGGTTCCTTT-3′
LINC01589	Forward Primer: 5′-AAATGGAATGCAGCCACACC-3′
	Reverse Primer: 5′-CCAAGAGGCCATCCGTCTTC-3′
NR2F1-AS1	Forward Primer: 5′-GGTCACGGAGAAAACAGGTTCA-3′
	Reverse Primer: 5′-CCCCAGAGCTGCATCCTTATG-3′
OSMR-AS1	Forward Primer: 5′-TTGGAAACCGAAAACTCGGC-3′
	Reverse Primer: 5′-ACATTGGGATGTTCTGCCCC-3′
PCAT1	Forward Primer: 5′-CCTCTAAGTGCCAGTGCAGG-3′
	Reverse Primer: 5′-ATGTATCTGCGCACCCTTTGA-3′
RP11-107N15.1	Forward Primer: 5′-GGGTCCTCAATGTGGGGTTT-3′
	Reverse Primer: 5′-TCGCTAGAGTCACCCCAGTT-3′
RP11-258F1.1	Forward Primer: 5′-CGTTGTACAGGCCCTTCTCA-3′
	Reverse Primer: 5′-GTGCGCACAACCCTGGTATC-3′
RP11-303E16.3	Forward Primer: 5′-CAGACTCCGTACGCCTTCAC-3′
	Reverse Primer: 5′-CTGAGCCTGCAACTCGACTG-3′
RP11-323N12.5	Forward Primer: 5′-TGGACCAGTCGAAACCCTTG-3′
	Reverse Primer: 5′-TCTCGACATCGAGGACCCAT-3′
RP11-326G21.1	Forward Primer: 5′-ACTCCGCATTACACCACTGA-3′
	Reverse Primer: 5′-CCCGAAACAGTACCAGGCAA-3′
RP11-346D6.6	Forward Primer: 5′-CAAGCAGCCCTGGAGAGTTTA-3′
	Reverse Primer: 5′-AACTTGGGGGTCACAGCATC-3′
RP11-373D23.3	Forward Primer: 5′-CTTCCAAGGCCCTGCATGAT-3′
	Reverse Primer: 5′-GGTGAGGGAAGACAACACGG-3′
RP11-379F4.4	Forward Primer: 5′-TGCCCGGTTTTATAGCCCTG-3′
	Reverse Primer: 5′-ATCTGTTCCGTGCTCCCTTC-3′
RP11-403A21.1	Forward Primer: 5′-AGGGATGGGGTCTCGAGTTT-3′
	Reverse Primer: 5′-TCAGCTGGTGGGTGTTTAGC-3′
RP11-465B22.8	Forward Primer: 5′-AGCCTGAGCTCATCCAACAC-3′
	Reverse Primer: 5′-GTGCGTGAACTGCAGACTTT-3′
RP3-483K16.4	Forward Primer: 5-′AGTTGCCATTGAGCTCCACAA-3′
	Reverse Primer: 5′-TGGACTACTGGCAGAAGCGT-3′
RP11-507M3.1	Forward Primer: 5′-CGCATTTTCCTGATTGGCCC-3′
	Reverse Primer: 5′-ACATTCCCCTTCAACGCCTG-3′
RP11-560J1.2	Forward Primer: 5′-CCTAGGGTAGTCCGAGGTCA-3′
	Reverse Primer: 5′ACAAAATACGCCCGGCAAAG-3′
RP11-61F12.1	Forward Primer: 5′-GGACGTGGTTTGCTAGGTGA-3′
	Reverse Primer: 5′-ACAGGTTTTCCGTCTCCGAC-3′
RP11-63G10.2	Forward Primer: 5′-ACCTGTGCCAGTGTGAACAA-3′
	Reverse Primer: 5′-GGGCTAGTCAAAGTCAGCGT-3′
SLC44A3-AS1	Forward Primer: 5′-AGCAACAGTGTAGTGGCGTA-3′
	Reverse Primer: 5′-CTGGCCTGTGATGCTTTTCC-3′
SNHG18	Forward Primer: 5′-CATGTTCCCAGAGGTTGGCA-3′
	Reverse Primer: 5′-AGAGGACAAGGCAAAACACTT-3′
TMEM220-AS1	Forward Primer: 5′-TCCAAGTCCCCTTCTGACTTC-3′
	Reverse Primer: 5′-CAGGCTCCTCAGGAAGAATCC-3′
SNORD3B-2	Forward Primer: 5′-GGCAGTGTAGCGAGAAAGGT-3′
	Reverse Primer: 5′-AATAGGAGGTGCCACACAGC-3′
RARRES1	Forward Primer: 5′-CGCTACAACCCAGAGTCTTTAC-3′
	Reverse Primes: 5′-TCACACTAGTGAGCTGTGCC-3′

### Knockdown of potential cis-acting SE-lncRNAs

150,000 DCIS and CA1 cells were plated into a six well plate using MFC10A media and transfected with 10 uL of 10 uM Gapmers or Antisense Oligonucleotides (ASOs) using 7.5 uL of RNAiMax Lipofectamine (Invitrogen Catalog No. 13778150). Cells were collected 48 h post-transfection and RNA was isolated using miRNeasy Mini Kit (Qiagen Catalog No. 217004). Reverse transcription of the collected RNA was performed using Applied Biosystems High-Capacity cDNA Reverse Transcription Kit (Life Technologies Catalog No. 4368813). RT-qPCR was performed using the primers mentioned above to analyze expression of the SE-lncRNAs and their neighboring mRNA after knockdown. U6 small nucleolar RNA was used as control for miR-200b expression while 18 s, Actin-B and GAPDH were used as controls to assess knockdown of the SE-lncRNAs and expression of RARRES1 mRNA.

SE-lncRNA LNA Gapmers:

**Table:** 

RP11-379F4.4:	Gapmer 1: 5′-ACTAGGTCCGAGGCAA-3′ (Qiagen Catalog No. 339511 LG00247071)
	Gapmer 2: 5′-ATGACTAAGGAACTAG-3′ (Qiagen Catalog No. 339511 LG00247084)
RP11-465B22.8:	Gapmer 1: 5′-GCGGTGAGGAGGTGCT-3′ (Qiagen Catalog No. 339511 LG00247067)
	Gapmer 2: 5′-GTGCGTGAACTGCAGA-3′ (Qiagen Catalog No. 339511 LG00247383)

### Chromatin immunoprecipitation (ChIP)

MCF10A, MCF10A-AT1, DCIS.com, and MCF10A-CA1 cells were grown to a final count of 5 × 10^6^. Cells were chemically crosslinked by the addition of 1 mL of fresh 10% formaldehyde solution for 10 min at room temperature on a rocker. After 10 min 1/10 volume of 1.25 M Glycine was added to quench unreacted formaldehyde and incubated for 5 min on rocker. Cells were pelleted at 1000 g for 5 min, washed twice with 1 × PBS, flash frozen in liquid nitrogen, and stored at − 80 °C prior to use. Cells were resuspended, lysed in lysis buffer (50 mM HEPES, 140 mM NaCl, 1 mM EDTA, 1% Triton X-100, 0.1% Sodium deoxycholate, 0.1% SDS), and sonicated to solubilize and shear crosslinked DNA. Sonication conditions vary depending on cells, but cells were sonicated using a Diagenode Bioruptor Sonicator and sonicated at power 7 for 13 × 30 s pulses (30 s pause between pulses) at 4 °C while samples were immersed in an ice bath. The sonicated cells were centrifuged for 10 min at 8000 g at 4 °C and the supernatant collected to proceed with immunoprecipitation. The resulting whole-cell extract volume was divided into two, one for IgG and the other for H3K27ac targeting. The samples were diluted in 1:10 ratio with RIPA buffer (50 mM Tris-HCl pH8, 150 mM NaCl, 2 mM EDTA pH8, 1% NP-40, 0.5% Sodium Deoxycholate, 0.1% SDS) incubated overnight at 4 °C with the 2.5 µg of the appropriate antibody, Cell Signaling IgG (Rabbit (DA1E) mAb IgG XP® Isotype Control #3900) and Abcam H3K27ac (Anti-Histone H3 (acetyl K27) antibody—ChIP Grade (ab4729)). The following day, 60 µL of ChIP Grade Protein G Magnetic beads (Cell Signaling 9006S) were washed three times with RIPA buffer and 30 µL each of the washed beads were added to IgG and H3K27ac samples and left rotating at 4 °C for 3 h. IgG and H3K27ac samples with magnetic beads were then washed three times with low salt wash buffer (0.1% SDS, 1% Triton X-100, 2 mM EDTA, 20 mM Tris–HCl pH 8.0, 150 mM NaCl) and one time with high salt wash buffer (0.1% SDS, 1% Triton X-100, 2 mM EDTA, 20 mM Tris-HCl pH 8.0, 500 mM NaCl). DNA was then eluted off the beads for each sample by heating at 65 °C at 1200 g for 1 h, cooling each sample at room temp for 2 min, centrifuging for 1 min at 10,000 g, and putting each sample on a magnet for 2 min and removing the liquid. 4.8 µL of 5 M NaCl and 2 µL RNase A (10 mg/mL) was added to each sample and incubated while shaking at 1200 rpm, 65 °C overnight. The next day, 2 µL proteinase K (20 mg/mL) was added to each sample and incubated while shaking at 1400 rpm, 60 °C for 1 h. DNA was purified using a QIAGEN QIAquick PCR purification kit (cat. Number 28104).

### ChIP-sequencing sample preparation and analysis

1 to 10 ng of DNA was prepared for sequencing using NEBNext Ultra II DNA Library Prep Kit (E7645S) and NEBNext® Multiplex Oligos for Illumina® (Index Primers Set 1) (E7335S). DNA was sequenced at the UAlbany Center for Functional Genomics using the Illumina NextSeq 500 with single end 75 bp reads. Quality of samples for ChIP was assessed using the Bioconductor package FastQC (version 0.11.9). Data were mapped to the human reference genome (hg38 assembly) using STAR Aligner (version 2.7.0). PCR duplicated reads were filtered using Sambamba (version 0.7.1). The MACS2 (version 2.2.7.1) algorithm was used to identify enriched regions (peaks). Default parameters were used with q-value of 0.05 except to ensure broad regions were identified; broad peak calling was added with a broad cutoff of 0.05. Reads were normalized to mapped reads.

### Super-enhancer identification

Super-enhancers were defined by stitching peaks using Rank Order Super-Enhancer (ROSE) (version python 2.7.3) with default parameters, except TSS exclusion zone size was adjusted to 250 bp. TSS exclusion was used because the H3K27ac signal is enriched for both active enhancers and promoters. However, several high-throughput reporter studies in mammals assessing either selected genomic regions (e.g., open chromatin regions or transcription factor binding sites) or human whole genomes have also found a substantial proportion of enhancers overlapping TSS-proximal regions [18, 19]. Signals for super-enhancers identified in each cell line were quantified in progression. Briefly, super-enhancer bed files for each cell line were collapsed into AllSEs.bed using bedtools merge. Coverage of AllSEs.bed was quantified using bam files for each cell line in the progression series using bedtools intersect. Reads were normalized to the sequencing depth, and fold-change was calculated. H3K27ac ChIP seq data for ER + patients was obtained from European Nucleotide Archive under project no. PRJEB22757 and for TNBC patient samples and TNBC cell lines under project no. PRJEB33558.

### Acquired/lost super-enhancer identification

Acquired super-enhancer regions were classified at the AT1 stage in progression by comparing super-enhancers ranked in AT1 cells with super-enhancers ranked in MCF10A cells and only keeping those that were ranked as super-enhancers in AT1 but not in MCF10A cells using bedtools intersect. Similarly, super-enhancers acquired at the DCIS stage were those that were ranked in DCIS cells but were not ranked in MCF10A and AT1 cells. Lastly, super-enhancers acquired at the CA1 stage were only ranked in CA1 cells but not in MCF10A, AT1, and DCIS cells. Lost super-enhancers were identified by comparing super-enhancers ranked in AT1, DCIS, and CA1 cells with super-enhancers in MCF10A cells and observing those that were not present in the corresponding cells but were in the normal MCF10A cells. Genes within 50 kb of the acquired/lost super-enhancers were classified using bedtools *closest* function. Gene Ontology (GO) analysis of these genes was performed using ENRICHR.

### Statistical analysis

All experiments were run in triplicate, except ChIP seq, which were done in duplicates. Data are represented as the mean ± standard deviation (mean ± SD). All statistical analyses were carried out using GraphPad Prism 9 Software (La Jolla, CA, USA). Statistical significance for SE-lncRNAs and mRNAs expression in our progression series were characterized by one-way ANOVA with Tukey correction. All significant and non-significant results are shown in Additional file: 7 Table S4. Expression of 14 target SE-lncRNAs in FFPE DCIS and IDC patient samples was analyzed by unpaired Student’s *t*-test and *p*-values are listed below in Fig. 4d and e for our two promising targets, while rest are shown in Additional file 2: Figure S2. Paired Student’s *t*-test was used to analyze knockdown of the two promising SE-lncRNAs as well as the corresponding expression of their associated mRNA, *p* < 0.05 (compared with the control antisense oligonucleotide) was considered significant and is marked with an asterisk in the figures.

## Results

### Global analysis of SE-lncRNAs acquired in IDC progression

**Fig. 1:**
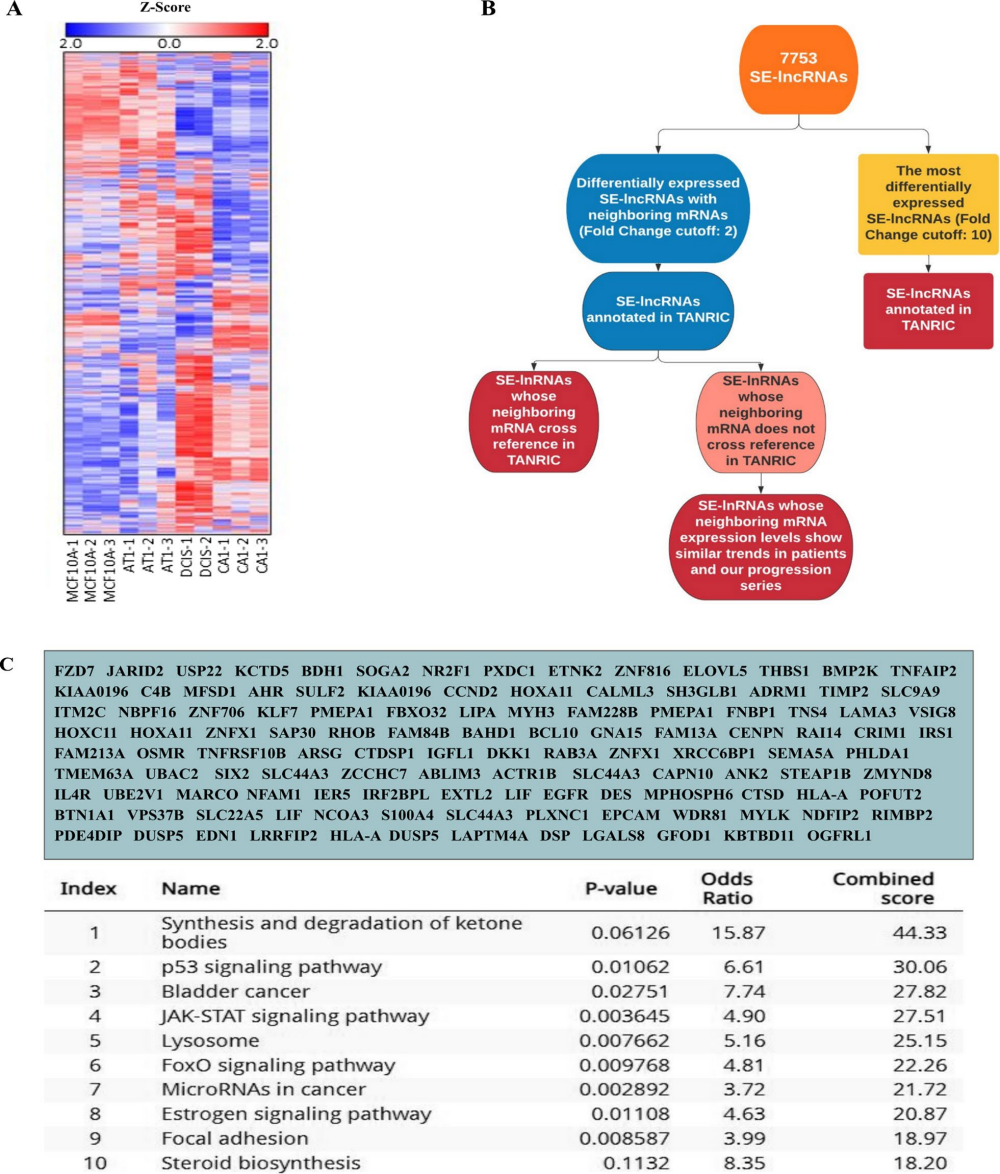
Filtering process taken to identify potentially *cis*-acting SE-lncRNAs from 7753 SE-lncRNAs that may contribute to progression to early-stage breast cancer. **a** Heatmap of 7753 SE-lncRNAs in MCF10A progression series. Hierarchical Clustering was performed. **b** Process to identify potentially *cis*-acting SE-lncRNAs crucial for progression of DCIS to IDC. Red boxes represent SE-lncRNAs that made the “cut.” **c** Gene Ontology analysis on the list of 138 mRNAs that were neighboring differentially expressed SE-lncRNAs (Fold Change cutoff: 2)

Underlying mechanisms that support breast cancer progression have been well studied. However, clear functional determinants segregating non-invasive from invasive tumors have yet to be defined. Acquired lncRNAs transcribed from super-enhancer loci can lead to discovering markers of progression, improving breast cancer diagnostics and treatment for patients. The MCF10A progression series mimics progression of breast cancer originating within the epithelial cells of the mammary ducts. The progression series was originally generated from MCF10A (10A) cells, a spontaneously immortalized mammary epithelial cell line derived from benign breast tissue from a woman with fibrocystic disease [20]. MCF10A cells were transformed with oncogenic HRAS to generate MCF10AT1 pre-malignant cells that form atypical ductal hyperplasia in mice [20]. MCF10DCIS.com (DCIS or DCIS.com) cells were derived from MCF10AT1 xenograft model and form predominantly comedo DCIS when injected into mice [21]. MCF10CA1 (CA1) cells, derived from MCF10AT1, form poorly differentiated malignant tumors in xenograft models [22]. Arraystar has developed a platform to comprehensively study super-enhancer lncRNAs and their downstream targets [23]. Taking advantage of this commercially available approach, we assessed SE-lncRNA expression within the MCF10A progression series. This analysis interrogated 7753 SE-lncRNAs (Fig. 1a), as well as 7040 associated mRNAs [24].

We found that super-enhancer associated lncRNAs are dynamically expressed during breast cancer progression. There are patterns of both SE-lncRNAs being acquired and lost as well as several SE-lncRNAs that exhibit a stepwise gain or loss in expression in progression (Additional file 4: Table S1). While the heat map from our analysis (Fig. 1a) demonstrates dynamic expression changes, the majority of these lncRNAs are not functionally defined.

Enhancer elements may become engaged either distally or locally to impact target gene expression. *Trans*-acting lncRNAs are transcribed, processed, and then vacate their sites of transcription to exert their function elsewhere, akin to mRNAs. Their final destination, be it in the cytoplasm or nucleus, does not depend on their transcription site [25]. By contrast, *cis*-acting lncRNAs are those whose activity is based at and dependent on the loci from which they are transcribed. Transcripts with the potential of acting in *cis* likely make up a substantial portion of known lncRNAs: the majority of lncRNAs are enriched in the chromatin fraction, and specifically are tethered to chromatin, presumably at their sites of transcription, through Pol II [25, 26]. If functional, this would indicate that the effects of these lncRNAs are centered at these loci. In addition, the fairly low levels at which lncRNAs are generally expressed, oftentimes just a few molecules per cell, naturally favor a *cis* mechanism of action, as diffusion or transport to other cellular compartments would render these transcripts too diluted to mediate a plausible function [27]. Identifying distally engaged enhancer elements is difficult since within the three-dimensional structure of the nucleus there are a myriad of possible locations they can interact.

To classify super-enhancers that may become locally engaged and regulate cancer progression, we applied filters to identify potential *cis*-acting SE-lncRNAs (Fig. 1b). First, from the 7753 SE-lncRNAs screened (Fig. 1b), we sorted and extracted SE-lncRNAs that are up or down regulated from normal 10A cells to invasive CA1 cells (fold change cut-off: ± 2). Furthermore, from these SE-lncRNAs that met our cutoff, we identified those which have neighboring genes within 50 kilo-bases upstream or downstream of the lncRNA that also demonstrate changing expression. The 50 kb window upstream and downstream were assigned as enhancers tend to loop to and associate with adjacent genes in order to activate their transcription [28] and primarily these interactions occur within a distance of ∼ 50 kb of the enhancer locus [29]. This allowed us to identify 138 SE-lncRNAs (Additional file 5: Table S2). Gene Ontology (GO) assessment on the list of mRNAs was performed to provide insight into what pathways the SE-lncRNAs might be regulating (Fig. 1c). Many pathways that appear in our GO analysis, such as focal adhesion, p53 signaling pathway, and JAK-STAT signaling pathway, constitute a major group of related signaling pathways that control proliferation, survival, angiogenesis, and metastasis of breast cancer, suggesting these SE-lncRNAs could be regulating canonical cancer promoting genes.

### Identifying clinically relevant potentially *cis*-acting SE-lncRNAs

**Fig. 2:**
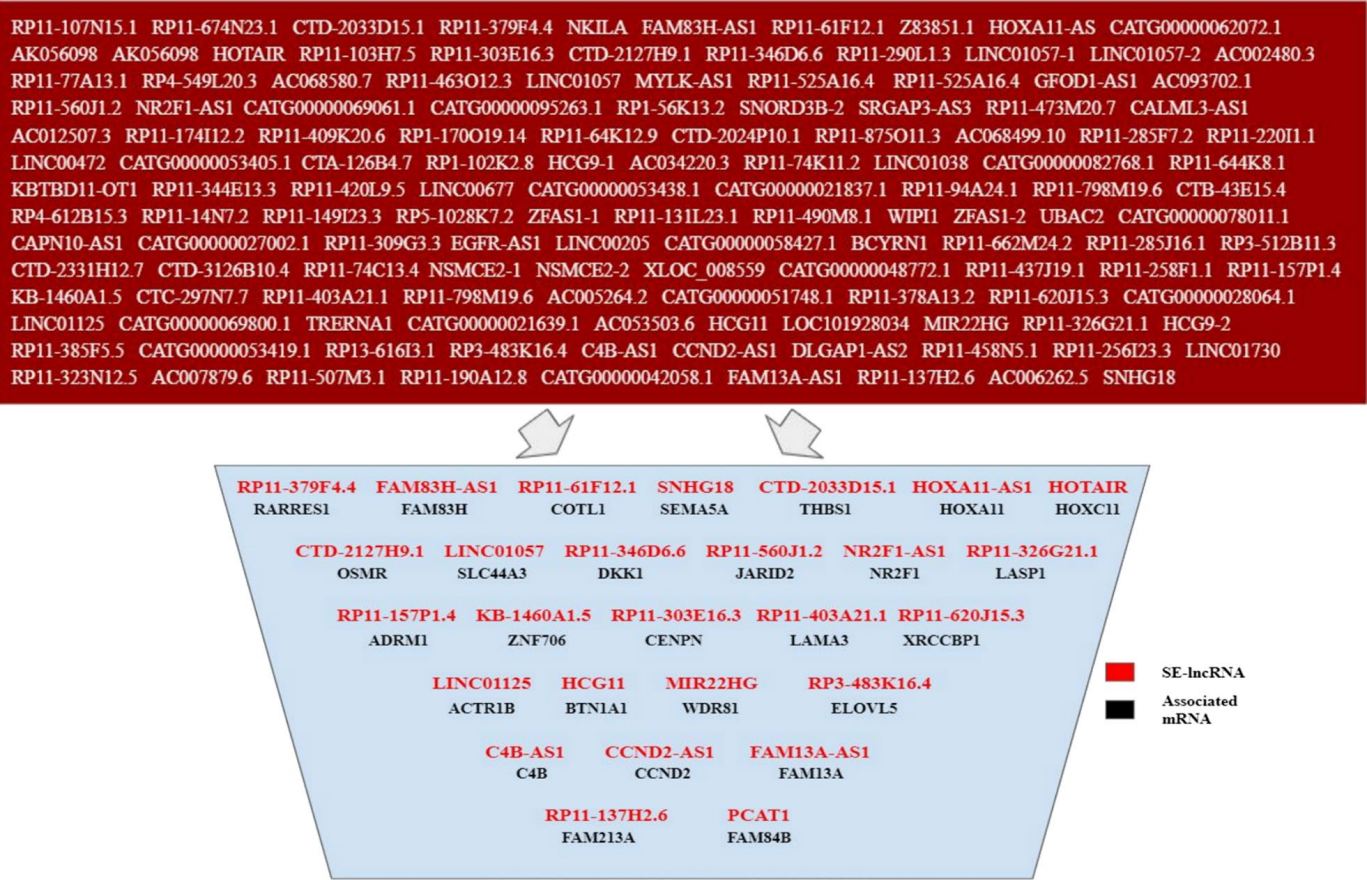
138 SE-lncRNAs filtered to 27 SE-lncRNAs and their neighboring mRNAs. From 138 SE-lncRNAs, 27 potentially *cis*-acting SE-lncRNAs and their neighboring mRNA were highlighted

**Fig. 3:**
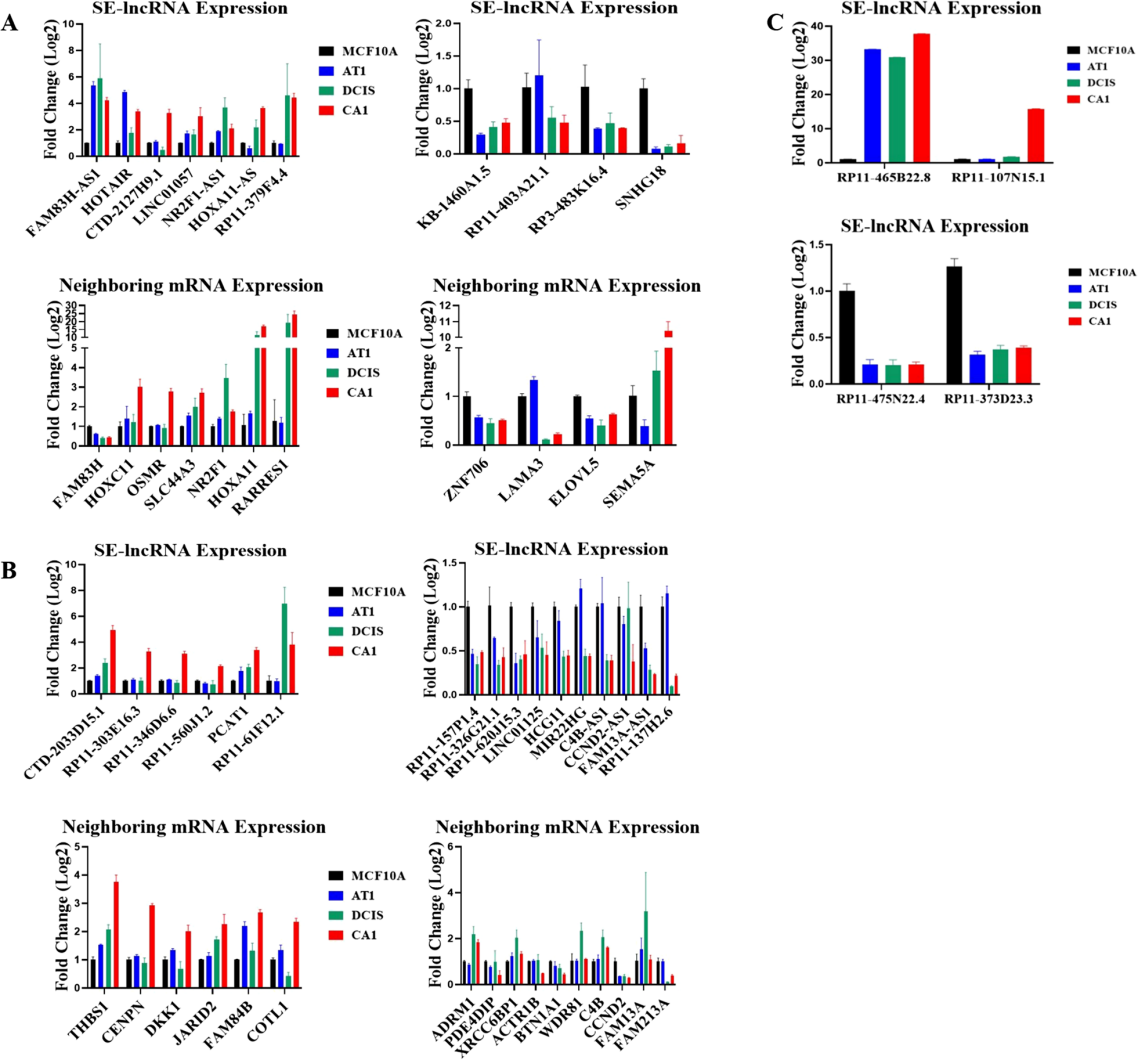
Expression of 27 potentially *cis*-acting SE-lncRNAs and their neighboring mRNA and 4 of the highest differentially expressed SE-lncRNAs in the MCF10A progression series. **a** Expression of 11 SE-lncRNAs and their neighboring mRNAs in progression that correlated in TANRIC. Top are upregulated SE-lncRNAs and their associated mRNA while bottom are down regulated and their associated mRNA. SE-lncRNA-mRNA pairs: FAM83H-AS1 and FAM83H, HOTAIR and HOXC11, CTD-2127H9.1 and OSMR, LINC01057 and SLC44A3, NR2F1-AS1 and NR2F1, HOXA11-AS and HOXA11, RP11-379F4.4 and RARRES1, KB-14601.5 and ZNF706, RP11-403A21.1 and LAMA3, RP3-483K16.4 and ELOVL5, SNHG18 and SEMA5A. **b** Expression of 16 SE-lncRNAs and their neighboring mRNAs in progression that did not correlate in TANRIC. Top are upregulated SE-lncRNAs and their associated mRNA, while bottom are down regulated and their associated mRNA. SE-lncRNA-mRNA pairs: CTD-2033D15.1 and THBS1, RP11-303E16.3 and CENPN, RP11-346D6.6 and DKK1, RP11-560J1.2 and JARID2, PCAT1 and FAM84B, RP11-61F12.1 and COTL1, RP11-57P1.4 and ADRM1, RP11-326G21.1 and PDE4DIP, RP11-620J15.3 and XRCC6BP1, LINC01125 and ACTR1B, HCG11 and BTN1A1, MIR22HG and WDR81, C4B-AS1 and C4B, CCND2-AS1 and CCND2, FAM13A-AS1 and FAM13A, RP11-137H2.6 and FAM213A. **c** Expression of the highest differentiated SE-lncRNAs in progression. Top two are upregulated SE-lncRNAs, while bottom two are down regulated. One-way ANOVA with Tukey correction was carried out to evaluate statistical significance of gene expression between cell lines, *n* = 3, * = *P* < 0.05, error bars represent standard deviation. Full statistical analysis is presented in Additional file 6: Table S3

To give clinical relevance to our data, these 138 SE-lncRNAs were cross-referenced with The Atlas of Non-coding RNAs In Cancer (TANRIC), which compiles patient data from The Cancer Genome Atlas (TCGA), and data from Cancer Cell Line Encyclopedia (CCLE) [30]. From our list of 138 SE-lncRNAs that are associated with mRNAs within the MCF10A series, we identified 27 SE-lncRNAs that are annotated in patient samples within TANRIC (Fig. 2) (Additional file 6: Table S3). If a SE-lncRNA is *cis*-acting and impacting enhancer activity or associated mRNA expression, then a change in SE-lncRNA expression should be accompanied by a change in target mRNA expression. Therefore, we next identified mRNAs whose expression levels correlated in patient samples within all subtypes of breast cancer for each of these 27 SE-lncRNAs (Pearson correlation coefficient ≥ 0.5 or ≤  − 0.5) (Additional file 6: Table S3). This analysis identified 11 SE-lncRNAs as potentially *cis*-acting (Fig. 3a). The remaining 16 SE-lncRNAs were further filtered out to focus on those whose neighboring mRNAs expression showed similar trends from normal to tumorous cells in patient samples and our progression series (Fig. 3b) (Additional file 6: Table S3). Lastly, we took the most differentiated SE-lncRNAs (Fig. 1b) within the progression series from our array (fold change cut-off: ± 10) and highlighted 4 that were annotated by TANRIC (Fig. 3c). One-way ANOVA with Tukey correction was performed to gauge statistical significance in expression changes in progression for all SE-lncRNAs presented in Fig. 3 and are reported in Additional file 7: Table S4. From our approach to identify potentially *cis*-acting SE-lncRNAs, we narrowed our list from 7753 to 31, with 27 promising targets as well as 4 SE-lncRNAs selected for follow up due to high differential expression within disease progression.

### Characterization of potential *cis*-acting SE-lncRNAs

**Fig. 4:**
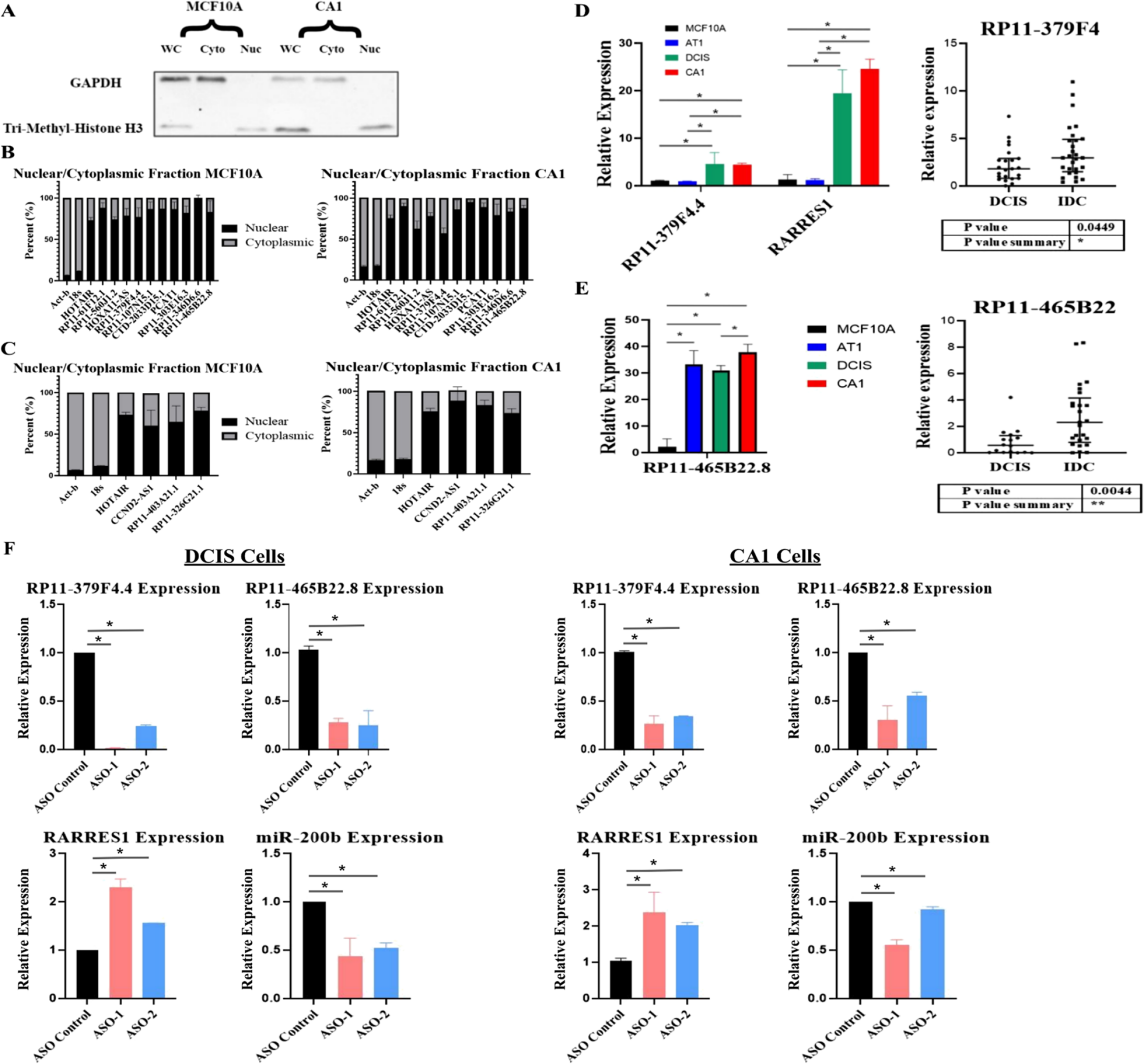
Localization of the potential *cis*-acting SE-lncRNAs. **a** Immunoblot of Cell Fractionation of Whole Cell Lysate, Cytoplasmic Fraction, and Nuclear Fraction in MCF10A and CA1 cells. GAPDH was used as control for Cytoplasmic fraction, while Tri-methyl Histone was used as control for Nuclear Fraction. **b, c** Localization of 14 SE-lncRNAs (11 up-regulated and 3 down-regulated) from our list of 27 potentially *cis*-acting SE-lncRNAs and 4 highest differentiated that are primarily localized within the nucleus. **d** Expression level of SE-lncRNA, RP11-379F4.1, and its neighboring mRNA, RARRES1, in MFC10A progression series, *n* = 3, * = *P* < 0.05,, one-way ANOVA with Tukey comparison, error bars represent standard deviation. Expression levels of SE-lncRNA RP11-379F4.1 in 24 DCIS and 24 IDC patients (* = *P* < 0.05), unpaired t test. **e** Expression level of the highest differentiated SE-lncRNA, RP11-465B22.8, in MCF10A progression series, *n* = 3, * = *P* < 0.05, one-way ANOVA with Tukey comparison, error bars represent standard deviation. Expression levels of SE-lncRNA RP11-465B22.8 in 16 DCIS and IDC patients (** = *P* < 0.005), unpaired t test. **f** Knockdown of the two target SE-lncRNAs was performed and expression of the SE-lncRNAs and their neighboring mRNAs was determined 48 h post-transfection in DCIS and CA1 cells, n = 3, * = *P* < 0.05, paired t-test, error bars represent standard deviation

Our approach to identify *cis*-acting SE-lncRNAs in progression provided 27 potential targets, 11 of which are highly promising, as well as 4 SE-lncRNAs with the highest differential expression within disease progression. *Cis*-acting SE-lncRNAs are expected to be localized within the nucleus; thus, to focus and narrow our list of potentially *cis-*acting SE-lncRNAs that are active in progression of DCIS to IDC, we investigated their sub cellular location. Whole cell lysate, as well as separated cytoplasmic fraction and nuclear fraction were extracted from the 10A and CA1 cells. These cell lines were chosen to determine if localization changed during progression. For all samples, 87.5% of the sample was used for RNA extraction, while 12.5% was used for an immunoblot to ensure the fractionation was done correctly (Fig. 4a). The 7:1 ratio was used to maximize RNA content as lncRNAs are relatively lowly expressed. From the 31 SE-lncRNAs, 14 were primarily localized within the nucleus (Fig. 4b, c). The remaining 17 were either localized within the cytoplasm or were expressed at low levels that localization could not be determined (Additional file 1: Figure S1).

Next, we utilized 24 DCIS and 24 IDC Formalin-Fixed Paraffin-Embedded (FFPE) patient samples of varying subtypes to assess the expression levels of the 14 SE-lncRNAs. Of the 14 SE-lncRNAs we tested in patient samples, RP11-379F4.4 and RP11-465B22.8 stood out as their increase in expression from DCIS to IDC was statistically significant and the expression levels matched our MCF10A model (Fig. 4d, e). The remaining targets either did not show significant results (Additional file 2: Figure S2) or expression was not determined. Albeit RP11-465B22.8 was not identified with an associated mRNA, the fact that it was the most up-regulated in progression prompted us to scan 50 kilobases upstream and downstream of the gene for potential target genes it might regulate. We discovered that this SE-lncRNA neighbors the miR-200 family of genes of which miR-200b is well studied to have a role as a tumor-suppressor [31] and, in other cases, a tumor promoter [32]. Having narrowed our list to one potential *cis*-acting SE-lncRNA and one SE-lncRNA that was highest differentiated in our model, we performed knockdown of the two targets and observed the expression of their associated gene in DCIS and CA1 cells (Fig. 4f). A two-fold increase was seen in the expression of RARRES1 48 h after knockdown of RP11-379F4.4 in DCIS and CA1 cells. Conversely, about a two-fold decrease in expression was observed for miR-200b 48 h post knockdown of RP11-465B22.8. These results illustrate that RP11-379F4.4 and RP11-465B22.8 are involved in regulating the expression of their neighboring gene. Importantly, this result shows that the lncRNA transcripts themselves are involved in regulating their neighboring gene and not the act of transcription of the SE-lncRNA itself. We have discovered several SE-lncRNAs that exhibit dynamic expression in progression, but more importantly, our approach has identified 2 of the most promising, potentially *cis*-acting, target SE-lncRNAs (Fig. 4d, e) that, by regulating nearby gene expression, could be crucial in the progression of indolent DCIS to IDC.

### Classification of super-enhancers in breast cancer progression

**Fig. 5:**
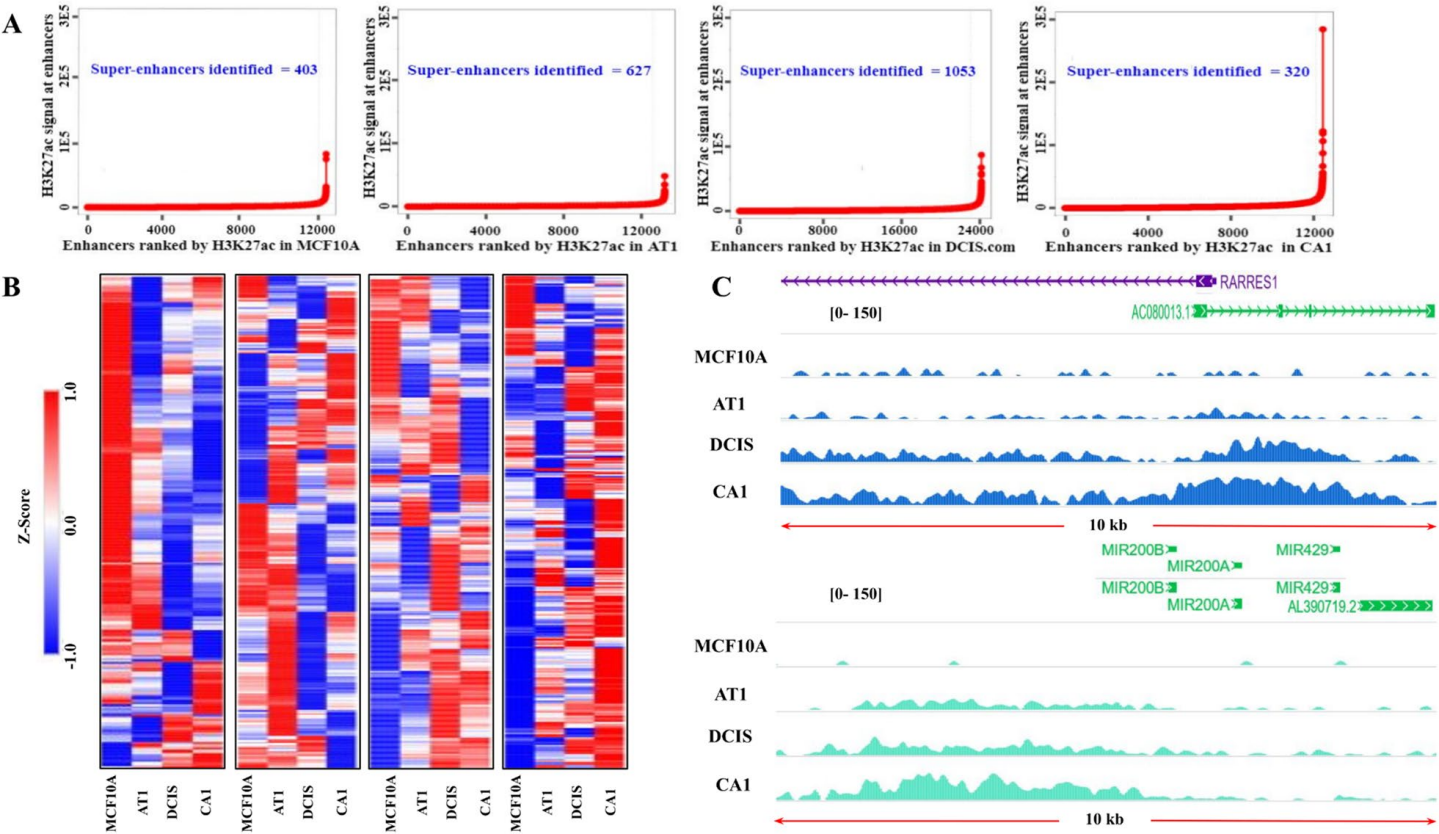
Super-enhancers Identified in MCF10A progression series. **a** Super-enhancers quantified in the MCF10A progression series by H3K27ac signal applying the ROSE Algorithm. **b** Heatmap of super-enhancer regions classified in each cell line in MCF10A progression series and their H3K27ac signal in corresponding cell lines in the series (From Left to Right: MCF10A super-enhancers, AT1 super-enhancers, DCIS.com super-enhancers, and CA1 super-enhancers). Hierarchical Clustering was performed. **c** H3K27ac signal at AC080013.1 (RP11-379F4.4), the most promising potential *cis*-acting SE-lncRNA, and AL390719.2 (RP11-465B22.8), the highest up-regulated SE-lncRNA in progression. H3K27ac signal was normalized to mapped reads

To complement our SE-lncRNA data, we identified super-enhancers in the MCF10A progression series. Enhancers are critical signaling elements regardless of their association with SE-lncRNAs, therefore we performed H3K27ac chromatin immunoprecipitation (ChIP) to identify global enhancer activity (Additional file 3: Figure S3). This analysis enabled us to identify super-enhancers at each stage of progression as well as active elements that may be predictive of progression. H3K27ac has previously been demonstrated to identify 2–3 times more enhancer regions of interest than Med1 alone [33]. Super-enhancer regions were identified using the Rank Order Super-Enhancer (ROSE) algorithm [34] (Fig. 5a). Our analysis determined 403 super-enhancer regions in 10A, 627 in AT1, 1053 in DCIS, and 320 in CA1 cells (Additional file 8: Table S5). Interestingly, a stepwise increase in the number of super-enhancer regions was seen from MCF10As to DCIS cells, however in invasive CA1 cells the number of super-enhancers regions classified decreased. Although the number of classified super-enhancers decreased in CA1 cells, the H3K signal is, on average, threefold more. In other words, even though the number of genomic regions that meet the threshold to be ranked as super-enhancers decrease from 10A to CA1 cells, a higher H3K signal is seen in CA1 cells indicating a higher expression of those genomic regions.

Despite being classified as a super-enhancer in one cell line, a region may not be classified as a super-enhancer in another because ROSE compares signal intensity within cell lines and not between cell lines to rank super-enhancers. Consequently, we analyzed H3K27ac signal intensity of super-enhancer regions identified in each cell line in the MCF10A progression series and observed their signal in the corresponding cell lines (Fig. 5b). Many of the super-enhancers classified in MCF10As lose signal intensity in AT1, DCIS, and CA1 cells indicating a loss of genomic expression within these regions. Similar trends of loss and gain of signal of the super-enhancers classified in AT1 and DCIS cells are seen (Fig. 5b). In conjunction with the higher H3K27ac signal observed for all CA1 identified super-enhancers, most of these regions are upregulated for H3K27ac signal (Fig. 5b). Correspondingly, we scanned the ranked super-enhancer regions in the progression series for our 2 identified SE-lncRNAs (Fig. 4d, e), RP11-379F4.4 and RP11-465B22.8. H3K27ac signal intensity was observed to increase in a stepwise manner for both targets (Fig. 5c), demonstrating that these genomic regions increase in accessibility in progression and contribute to a higher expression of their neighboring genes. Furthermore, RP11-379F4.4 is ranked as a super-enhancer in DCIS (#8) as well as CA1 (#92) cells (Additional file 8: Table S5). Though the region containing RP11-465B22.8 was not classified as a super-enhancer in our progression, it still exhibits higher H3K27ac occupancy in DCIS and CA1 cells when compared to normal. Specifically, the region upstream of RP11-465B22.8 and the miR-200 family is higher in H3K27ac signal intensity. Super-enhancer regions recruit transcription machinery and tend to loop to promoter regions of genes they regulate [35]. Hence, that region (and its H3K27ac occupancy) is consistent with the profile of an active enhancer involved in regulation of the expression of miR-200 family of genes with the help of RP11-465B22.8. These changes in the enhancer activation of DNA within progression are crucial to helping understanding progression of normal tissue to cancerous.

### Acquired/lost super-enhancers in breast cancer progression

**Fig. 6:**
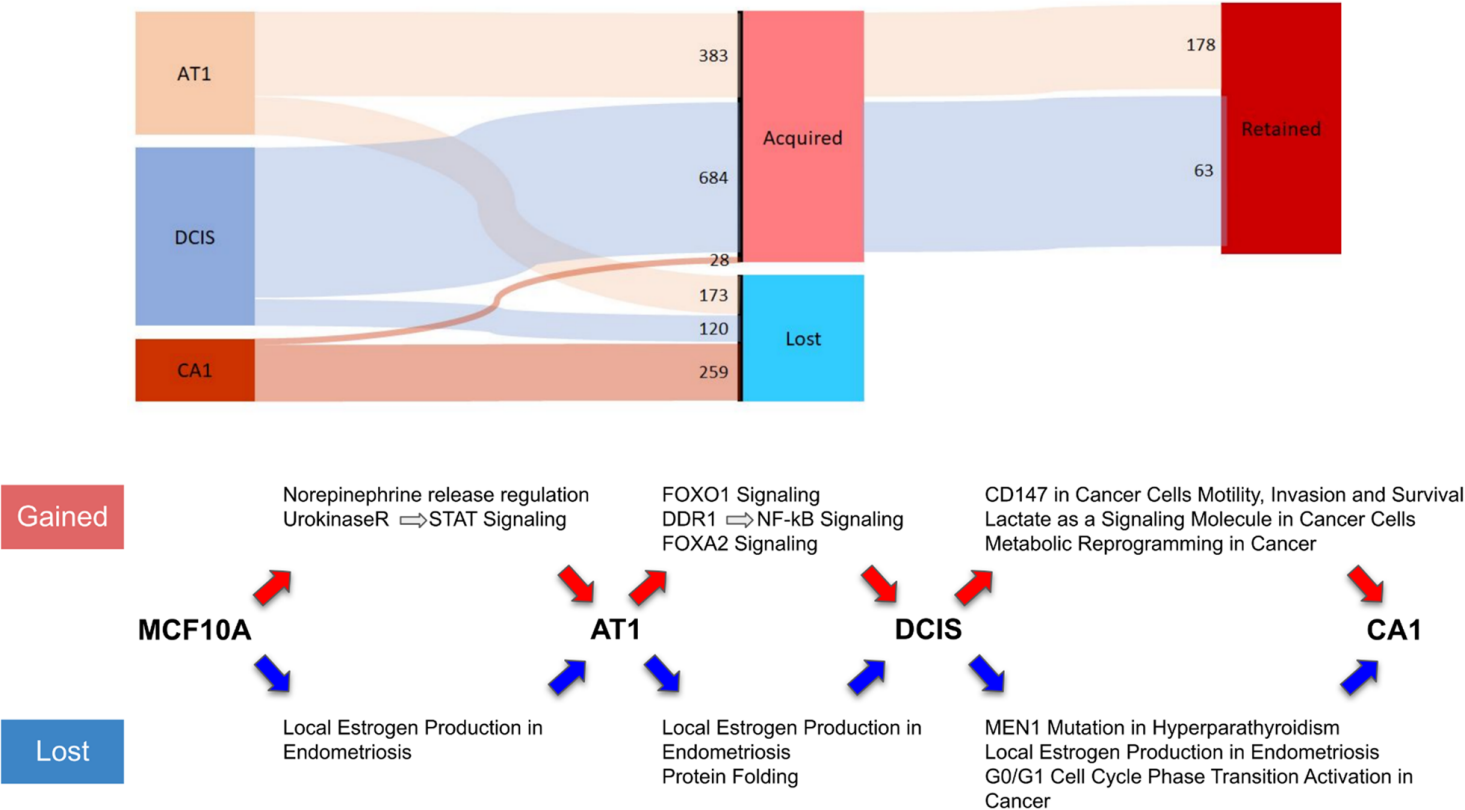
Super-enhancers acquired/lost in progression. Number of newly acquired super-enhancer at each stage in progression in the MCF10A progression series and the number of lost super-enhancer at each stage in progression relative to MCF10A. Super-enhancers that were acquired at a specific stage and were also ranked as super-enhancers in later stages up to CA1 cells were classified as retained. GO analysis of neighboring genes (50 kb up or downstream) for each list of acquired/lost super-enhancer regions classified in the MCF10A progression series. Pathways acquired in progression at each stage are indicated by red arrows. Pathways lost in progression at each stage are indicated by blue arrows

We examined our super-enhancer list to unravel newly acquired super-enhancers at each stage in progression in addition to super-enhancers lost at each stage relative to MCF10A cells (Fig. 6). 383 super-enhancers were newly acquired at the AT1 stage, 684 were newly acquired in DCIS, while only 28 were newly acquired at the CA1 stage (Additional file 8: Table S5). Consistent with previously established trends, most of the newly acquired super-enhancers were classified in AT1 and DCIS cells. Gene ontology assessment on the closest genes to these regions reveal many acquired pathways including STAT signaling in AT1 and NF-kB signaling in DCIS, which are putative pathways known for promoting proliferation and tumorigenesis (Fig. 6). Conversely, 173, 120, and 259 super-enhancers were lost at AT1, DCIS, and CA1 stages, respectively, (Additional file 8: Table S5). Similarly, a gene ontology assessment on genes (50 kb up or downstream (100 kb total) as enhancers tend to regulate genes within approximately a 50 kb distance upstream and downstream of its locus [29]) near lost super-enhancers reveal protein folding and local estrogen production as major pathways lost in progression (Fig. 6).

### Comparison of acquired/lost super-enhancers in the MCF10A model with super-enhancers classified in ER + and triple negative breast cancer patients

**Fig. 7:**
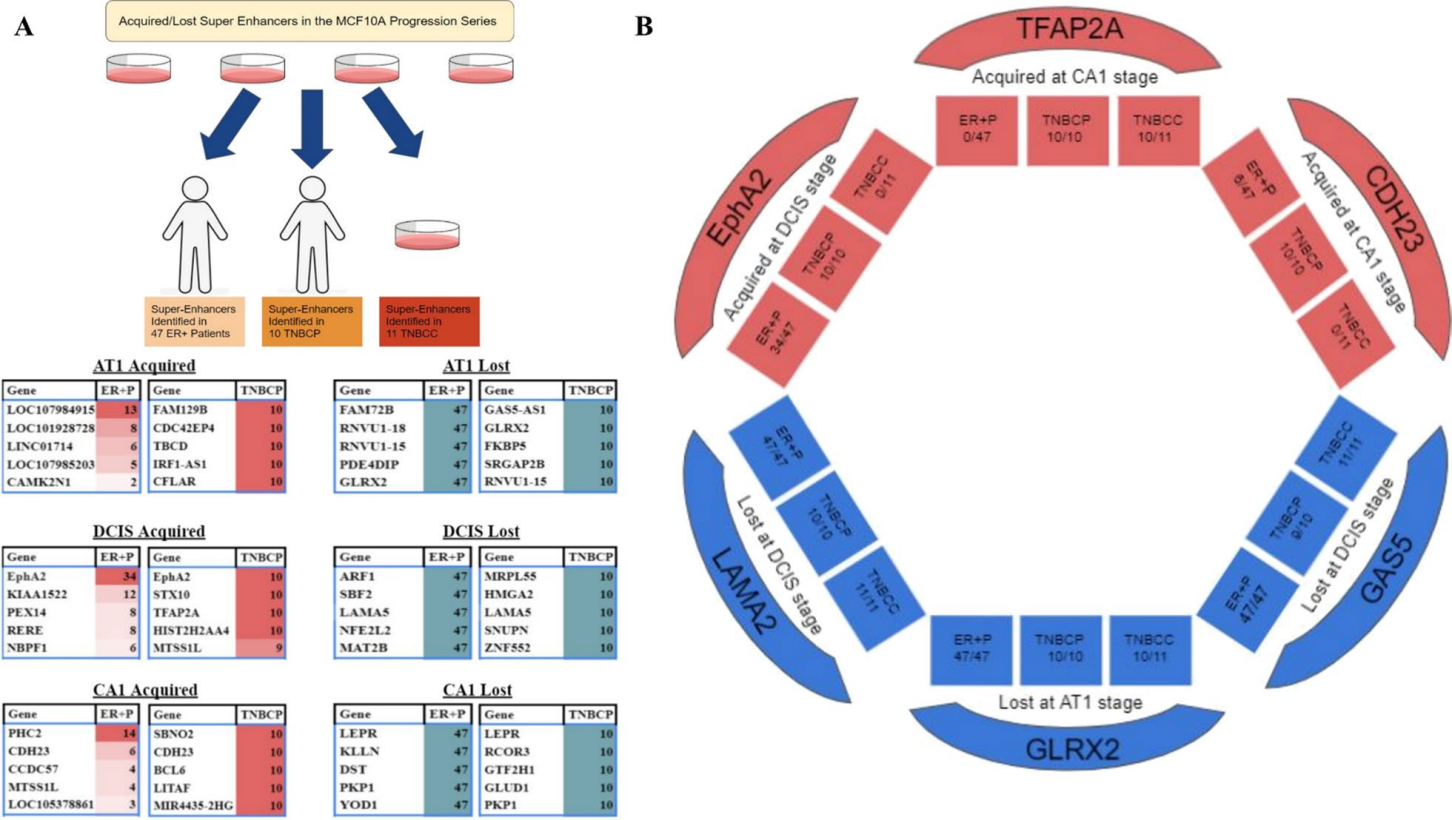
Comparison of Super-Enhancers Acquired/Lost in Progression with Super-Enhancers Identified in Patient Samples. **a** Schematic of the comparison between super-enhancers acquired/lost in progression with 47 estrogen receptor positive patients (ER + P), 10 triple-negative breast cancer patients (TNBCP) and 11 triple-negative breast cancer cell lines (TNBCC). Top super-enhancers regions within our comparison are represented by the mRNA nearest the super-enhancer. The stage super-enhancers were acquired/lost at within our progression series, how many patient samples they were present/not present, and the nearest mRNA are represented below the schematic. **b** 6 of the most interesting super-enhancer regions in our progression series that corresponded with patient samples with their nearest gene highlighted

To couple our MCF10A series findings with patient data, we identified super-enhancers in 47 estrogen receptor positive (ER +) patient samples [36], 10 triple-negative breast cancer (TNBC) patient samples, and 11 triple-negative breast cancer cell lines (TNBCC) [37] (Additional file 9: Table S6). Comparison analysis of acquired super-enhancer regions at each stage in progression was performed with super-enhancer regions in patient samples and TNBCC (Fig. 7a) (Additional file 9: Table S6). Super-enhancers acquired/lost in progression that were most represented or absent in ER + and TNBC patients are listed with respect to their nearest gene (Fig. 7a). We highlight super-enhancer regions and their neighboring genes that were well represented in patient samples, TNBCC, and our progression series (Fig. 7b). Ephrin type-A receptor 2 (EphA2) region was classified as a super-enhancer at the DCIS stage in progression as well as being classified as a super-enhancer region in 34/47 ER + patients and 10/10 TNBC patients. Similarly, the region containing Cadherin 23 (CDH23) which was acquired at the CA1 stage in progression as a super-enhancer, was classified as a super-enhancer in 6/47 ER + patients and 10/10 TNBC patients. Interestingly, Transcription Factor AP-2 Alpha (TFAP2A) region was not ranked as a super-enhancer in any of the ER + patients while it was in all TNBC patients. Sumoylation of TFAP2A has been shown to block its ability to induce the expression of luminal genes and maintain a basal/triple-negative cancer subtype [38]. At the same time, genomic regions containing Glutaredoxin 2 (GLRX2), Laminin Subunit Alpha 2 (LAMA2), and Growth Arrest Specific 5 (GAS5), which are lost at the AT1 and DCIS stages, respectively, were not ranked as super-enhancers in any of the patients or TNBC cell lines. GAS5 is a well-studied down regulated lncRNA in breast cancer while GLRX2 is a protein that localizes to the mitochondria where it functions in mitochondrial redox homeostasis and is important for the protection against and recovery from oxidative stress [39]. Considering metabolic reprogramming is one of the pathways acquired (Fig. 6), epigenetic changes within the GLRX2 region could play a crucial role in progression. Super-enhancers are central to driving expression of genes controlling cell identity and stimulating oncogenic transcription, thus, cancerous phenotype relies on these abnormal transcription propelled by super-enhancers. Here we have highlighted super-enhancers acquired/lost in progression and cross-referencing these regions with super-enhancers in patient samples, we unravel epigenetic changes driving cell identity and progression. Lastly, this validates results from our model with patient samples and current literature demonstrating a robust discovery platform.

## Discussion

Currently, functional determinants of DCIS progression to an invasive lesion are unknown[17]. This study profiles global SE-lncRNA expression in the MCF10A progression series giving insight into numerous SE-lncRNAs that are differentially expressed in progression. Furthermore, these SE-lncRNAs can play essential roles in transcriptional regulation through controlling SEs activity to regulate a broad range of physiological and pathological processes, especially tumorigenesis. Equally, SE-lncRNAs can regulate gene expression by affecting gene promoter activity. Although SE-lncRNAs significantly contribute to gene expression, the systematic identification of SE-lncRNAs and their regulated genes still lacks comprehensive recognition [40] [41]. Hence, this study also elucidates the expression levels of mRNAs associated with those SE-lncRNAs. In addition, using stringent and comprehensive set of filters that combined Cancer Cell line data with patient data, we have highlighted 27 potentially *cis-*acting SE-lncRNAs and their target mRNAs coupled with 4 SE-lncRNAs that are highest differentiated in disease progression. From this list we have identified RP11-379F4.4 (AC080013.1) as a promising *cis*-acting SE-lncRNA to its target gene Retinoic acid receptor responder element 1 (RARRES1). Interestingly, RARRES1 functions as an invasion suppressor. This function of the gene was confirmed in metastatic prostate cancer (CaP) cell line (PC3M) by Oldridge et al. [42]. Likewise, RARRES1 is able to increase Sirtuin 1, while it decreases the mechanistic target of rapamycin (mTOR), two important regulators of energy homeostasis. RARRES1 is differentially expressed in metabolic diseases and is associated with biological hallmarks that require metabolic reprogramming. Metabolic reprogramming is now considered a hallmark of cancer etiology [43]. Although RARRES1 is among the most commonly methylated genes in multiple cancers, it is increased in basal-like hormone receptor negative breast cancer and in liver cirrhosis, a risk factor for hepatocellular cancer [44].

Similarly, we also have highlighted RP11-465B22.8 (AL390712.1) as the most differentiated SE-lncRNA in progression. Comparatively, miR-200b, neighboring RP11-465B22.8, is part of the well-known tumor suppressor miR200 family. The family of miR-200 includes five members: miR-200a, miR-200b, miR-200c, miR-429, and miR-141. miR-200b, which acts as an antioncogene, participates in the proliferation and metastasis inhibition of different kinds of cancers by downregulating target molecules. For instance, miR-200b inhibition promotes Rac1 activation and increases the metastatic potential of HBEC cells [45]. miR-200b can repress angiogenesis by targeting angiogenic factors and receptors [46]. It can inhibit the epithelial to mesenchymal transition (EMT) by inactivating transcription factors in breast cancer. miR-200b is associated with the estrogen receptor status of breast cancer cells [46, 47]. Zheng et al. also highlight Fucosyltransferase IV (FUT4) could apply as a novel target for miR-200b that suppresses the proliferation and metastasis of breast cancer cells by reducing α1,3-fucosylation and LeY biosynthesis of glycoproteins [31]. Thus, SE-lncRNAs RP11-379F4.4 and RP11-465B22.8 and their respective potential targets are promising candidates for their *cis-*acting capabilities leading to progression. Further studies will have to be conducted to assess the mechanism of *cis* action for the SE-lncRNAs and how they promote DCIS lesions toward IDC.

In the past decade, increasing evidence has revealed that super-enhancers play a vital role in tumorigenesis and there is great interest in developing super-enhancer therapeutics, thus, this study also elucidates super-enhancers that are acquired or lost in progression [6]. We profiled H3K27ac using ChIP in the MCF10A progression series. As enhancers and super-enhancers play an important role in driving cell identity, the alteration in intensity of the H3K27ac in progression paints a picture of epigenetic changes which could be leading to the hijacking of genes involved in various hallmarks of cancer.

Correspondingly, we uncover newly acquired super-enhancers at each stage in progression in addition to super-enhancers lost at each stage relative to MCF10A cells. This analysis enabled us to highlight and distinguish loci that are activated/suppressed. Importantly, we identified 28 super-enhancers that are acquired from DCIS to CA1 transition and over 100 super-enhancers that are lost from DCIS to CA1. Furthermore, we classify genes neighboring these acquired/lost regions which identify pathways that contribute to progression. For example, STAT signaling is acquired in AT1 transition from normal cells, while NF-kB signaling is acquired in the transition to DCIS. Similarly, protein folding and local estrogen production pathways are lost overall in progression in addition to regions responsible for cell cycle regulation. These are canonical cancer pathways leading to proliferation and metastasis. Here we show how super-enhancers being activated/suppressed neighboring genes involved in these pathways play a pivotal role in their misregulation.

Comparison of acquired/lost super-enhancer regions with super-enhancer regions classified in 47 ER + patients, 10 TNBC patients, and 11 TNBC cell lines provides clinical relevance. This comprehensive analysis reveals epigenetic changes at the genome wide level in breast tumors. For example, Ephrin type-A receptor 2 (EphA2), is a receptor tyrosine kinase which binds ephrin-A family ligands residing on adjacent cells, leading to contact-dependent bidirectional signaling into neighboring cells. This gene has been known to regulate migration, integrin-mediated adhesion, proliferation, and differentiation of cells through DSG1/desmoglein-1 and inhibition of the ERK1/ERK2 signaling pathway [48]. EphA2 has been implicated in breast tumors and resistance of tumors to targeted therapies [48–50]. Targeting EphA2 has been shown to inhibit cell cycle progression and proliferation [48]. The genomic region containing EphA2 is an acquired super-enhancer at the DCIS stage in our progression model. Interestingly, it is also classified as a super-enhancer region in 34/47 ER + patient samples and 10/10 TNBC patients in our analysis. This explains the observed overexpression of this gene in breast tumors and can provide new targeting methods. Identically, the region containing GAS5 lncRNA is classified as a super-enhancer on normal MCF10A cells, however, is lost at the AT1, DCIS, and CA1 stages. Neither is it classified as a super-enhancer in all 47 ER + and 9/10 TNBC patients implying a mechanism of the down-regulation seen of this lncRNA in breast tumors. GAS5 can bind to the DNA binding domain of the glucocorticoid receptor inactivating it and subsequently inhibiting the regulation of its target genes [51]. In addition, GAS5 can regulate the transcriptional activity of other receptors, such as androgen and progesterone and has been suggested as a potential tumor suppressor due to its pro-apoptotic function [52]. The epigenetic changes occurring within this region can explain the down-regulation of this lncRNA and reduce its effects in carrying out normal processes leading to tumorigenesis.

Lastly, we also analyzed the regions that contained our two identified targets, RP11-379F4.4 and RP11-465B22.8, to see if they were classified as super-enhancers in progression. RP11-379F4.4 was ranked as a super-enhancer in DCIS as well as CA1 cells and is seen to be acquired during progression. RP11-465B22.8 was not ranked as a super-enhancer by the ROSE algorithm in any of the cells, however, the signal intensity of H3K27ac saw a dramatic stepwise increase from normal to CA1 cells. This result verifies the enhanced transcription that is observed of these SE-lncRNAs and hints at a possible role that they may play to induce progression. We also examined if RP11-379F4.4, which was an acquired super-enhancer at the DCIS stage in progression, was classified as a super-enhancer in any of the patient samples. Although RP11-379F4.4 was not classified as a super-enhancer in patient samples, its classification as an enhancer represents open DNA, and most likely, the cause of its higher expression seen in CA1 cells and patient tumors. Super-enhancers associate with key oncogenes in cancers and drive expression of genes that define cell identity. Additionally, cancer cells can acquire super-enhancers at oncogenes while losing super-enhancers at tumor suppressing genes. Understanding the alterations in the genomic landscape within breast tumors will uncover underlying biology that can be useful in diagnostic and targeted therapies.

The data presented here highlight several SE-lncRNAs that through their potential *cis*-acting abilities play an important role in progression of DCIS lesions into invasive IDC. Furthermore, we have identified two promising target SE-lncRNAs that may drive cancer progression through the regulation of their neighboring gene. Future studies will expand on understanding their potential *cis-*acting functions. Equally, we reveal acquired/lost super-enhancers in progression coupled with patient data that can help elucidate the epigenetic alterations promoting cancerous phenotypes. While there may not be one key protein that determines DCIS progression, understanding the networks of signaling pathways that change during progression unravel critical changes that push a DCIS lesion to be invasive.

## Conclusion

Altogether, this comprehensive study of breast cancer cell lines coupled with patient samples provides a unique platform that identifies differentially expressed SE-lncRNAs and acquired/lost super-enhancers in progression of breast cancer important for promoting DCIS lesions to IDC.

## Supplementary Information

**Additional file 1: Figure S1**: Localization of the potential cis-acting SE-lncRNAs. Localization of the 12 SE-lncRNAs from our list of 27 potentially cis-acting SE-lncRNAs and 4 highest differentiated that are primarily localized within the cytoplasm. Act-b and 18s are used as controls.

**Additional file 2: Figure S2**: Expression levels of potential cis-acting SE-lncRNAs in breast cancer patients. A) Expression levels of remaining up-regulated SE-lncRNAs in 24 DCIS and 24 IDC patients, * = P < 0.05. B) Expression levels of remaining down-regulated SE-lncRNAs in 24 DCIS and 24 IDC patients,* = P < 0.05. RP11-61F12 does show a statistically significant increase in expression from DCIS to IDC in patient samples, and this mimics our progressions series data. It has been marked for future studies; however, it is not the most promising of our target SE-lncRNAs.

**Additional file 3: Figure S3**: Specificity of H3K27ac ChIP antibody. Fold enrichment of the H3K27ac antibody between control promoter region of Myt1 and enhancer region of MYC.

**Additional file 4: Table S1**: Raw values of all SE-lncRNAs in the MCF10A progression series.

**Additional file 5: Table S2**: 138 SE-lncRNAs, their corresponding mRNAs, and the log2 fold change of each in the MCF10A progression series.

**Additional file 6: Table S3**: 27 SE-lncRNAs, their associated gene, and all mRNAs correlated within the TANRIC database with their Pearson Correlation Coefficient that met our threshold.

**Additional file 7: Table S4**: Statistical significance for SE-lncRNAs and their associated mRNAs expression in our progression series characterized by one-way ANOVA with Tukey correction. All significant and non-significant results are shown.

**Additional file 8: Table S5**: Super-enhancers identified using H3K27ac ChIP seq and classified by the ROSE algorithm in each cell line in the MCF10A progression series.

**Additional file 9: Table S6**: Super-enhancers identified in 47 ER+, 10 TNBC, and 11 TNBCC compared with super-enhancers identified in each cell line in the MCF10A progression series.

## Data Availability

The dataset generated and analyzed during the current study is available from the corresponding author on request. In addition, the H3K27ac ChIP-seq data for the MCF10A progression series discussed in this publication has been deposited in NCBI’s Gene Expression Omnibus database under accession number GSE181524.

## Abbreviations

DCIS: Ductal carcinoma in situ
IDC: Invasive ductal carcinoma
SE: Super-enhancer
e-lncRNA: Enhancer long non-coding RNA
SE-lncRNA: Super-enhancer long non-coding RNA
10A: MCF10A
AT1: MCF10A-AT1
DCIS: MCF10A-DCIS
CA1: MCF10A-CA1
ChIP: Chromatin immunoprecipitation
ASO: Antisense oligonucleotide
ER +: Estrogen receptor positive
TNBC: Triple negative breast cancer
