# Supplementary material for: Cis-acting super-enhancer lncRNAs as biomarkers to early-stage breast cancer
Source: Breast Cancer Res. 2021 Oct 30;23:101. doi: 10.1186/s13058-021-01479-8 (PMC8557595; doi:10.1186/s13058-021-01479-8)
Supplement: Supplementary file 1 — Additional file 1: Figure S1: Localization of the potential cis-acting SE-lncRNAs. Localization of the 12 SE-lncRNAs from our list of 27 potentially cis-acting SE-lncRNAs and 4 highest differentiated that are primarily localized within the cytoplasm. Act-b and 18s are used as controls. [file 13058_2021_1479_MOESM1_ESM.docx]

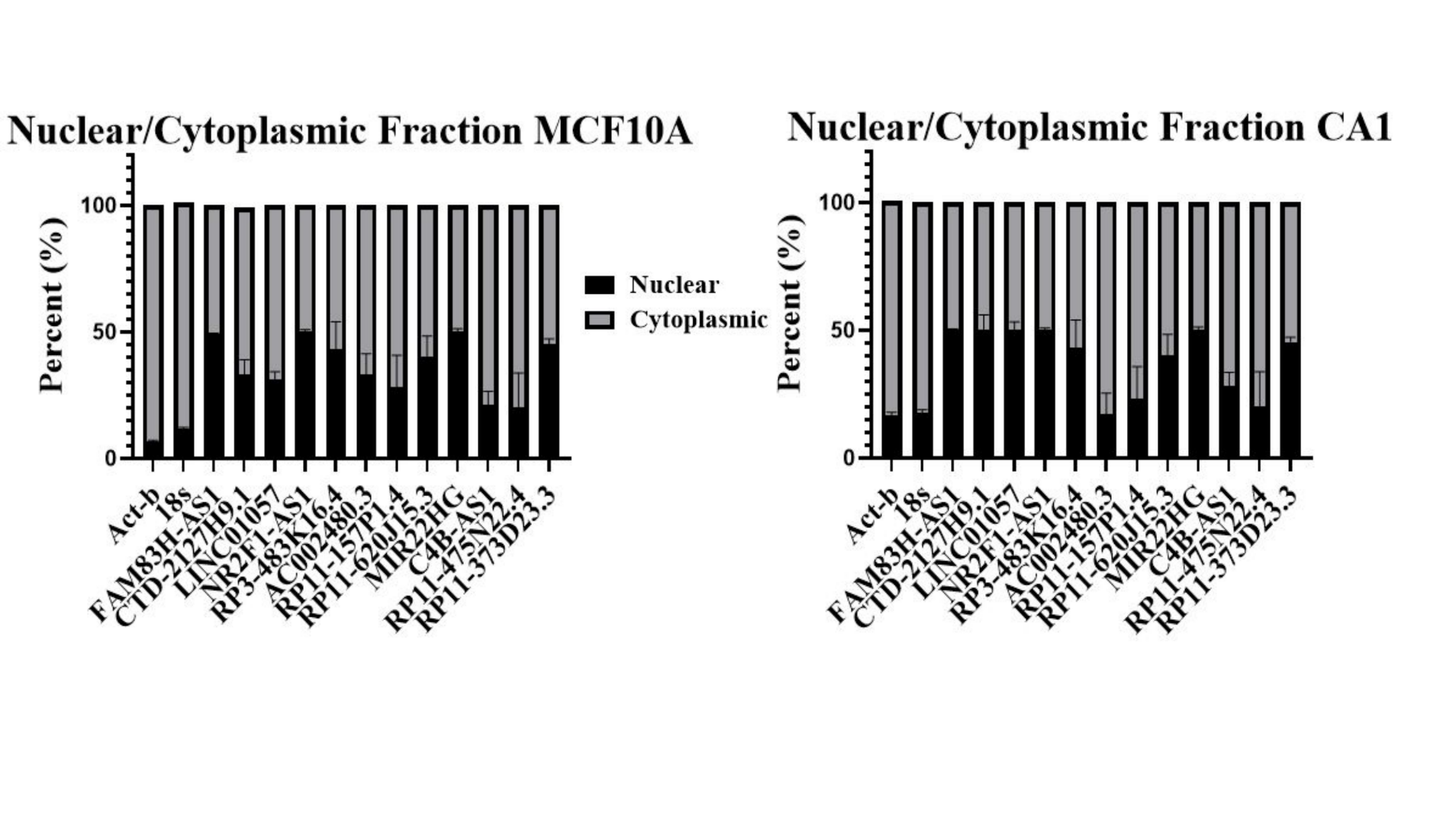


**Supplementary Figure 1: Localization of the potential *cis*-acting SE-lncRNAs**. Localization of the 12 SE-lncRNAs from our list of 27 potentially *cis*-acting SE-lncRNAs and 4 highest differentiated that are primarily localized within the cytoplasm. Act-b and 18s are used as controls.
