# Supplementary material for: Cis-acting super-enhancer lncRNAs as biomarkers to early-stage breast cancer
Source: Breast Cancer Res. 2021 Oct 30;23:101. doi: 10.1186/s13058-021-01479-8 (PMC8557595; doi:10.1186/s13058-021-01479-8)
Supplement: Supplementary file 2 — Additional file 2: Figure S2: Expression levels of potential cis-acting SE-lncRNAs in breast cancer patients. A) Expression levels of remaining up-regulated SE-lncRNAs in 24 DCIS and 24 IDC patients, * = P < 0.05. B) Expression levels of remaining down-regulated SE-lncRNAs in 24 DCIS and 24 IDC patients,* = P < 0.05. RP11-61F12 does show a statistically significant increase in expression from DCIS to IDC in patient samples, and this mimics our progressions series data. It has been marked for future studies; however, it is not the most promising of our target SE-lncRNAs. [file 13058_2021_1479_MOESM2_ESM.docx]

**
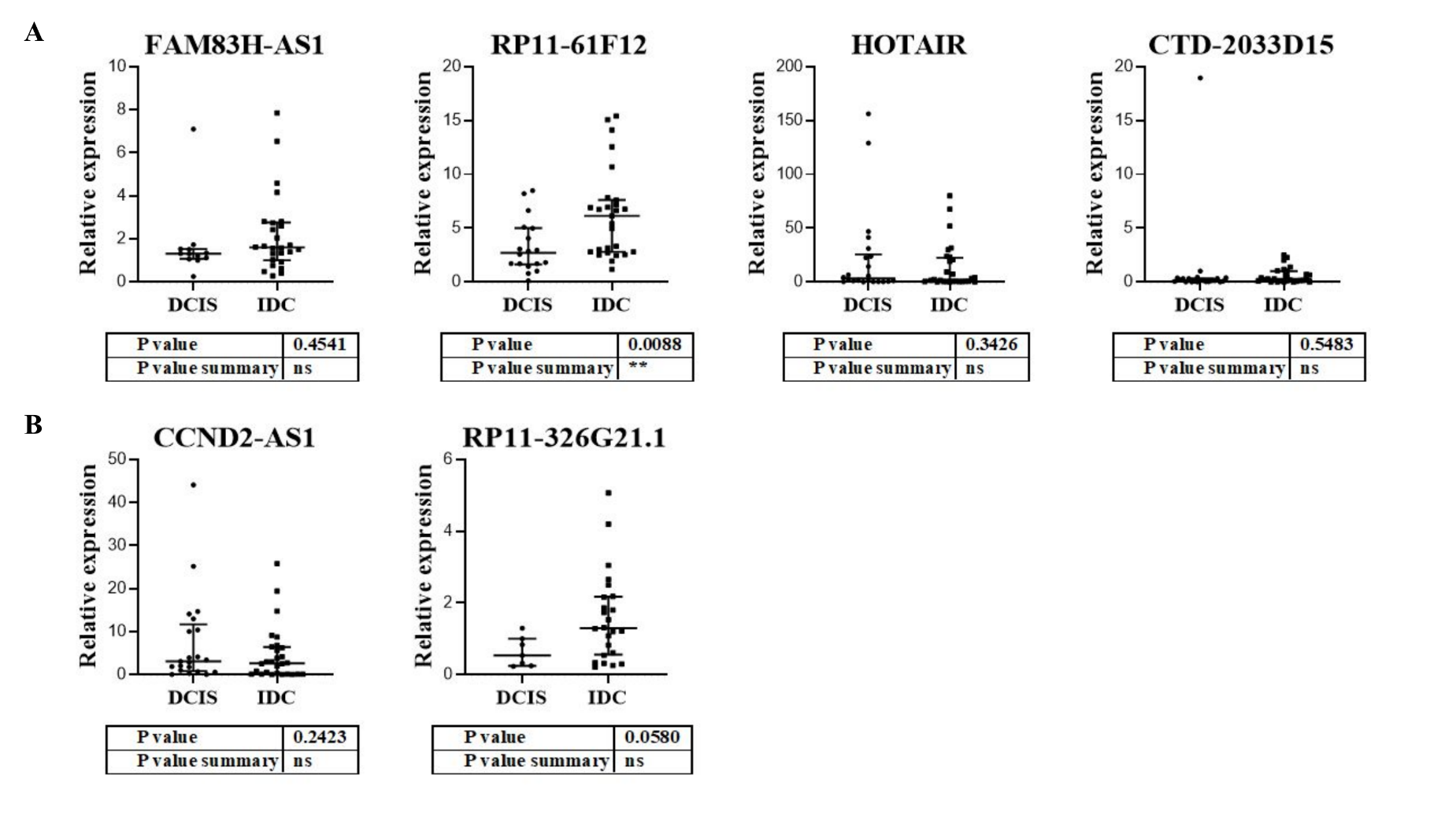
**

**Supplementary Figure 2: Expression levels of potential *cis*-acting SE-lncRNAs in breast cancer patients. A)**  Expression levels of remaining up-regulated SE-lncRNAs in 24 DCIS and 24 IDC patients, * = P < 0.05. **B)**  **)**  Expression levels of remaining down-regulated SE-lncRNAs in 24 DCIS and 24 IDC patients,* = P < 0.5. RP11-61F12 does show a statistically significant increase in expression from DCIS to IDC in patient samples, and this mimics our progressions series data. It has been marked for future studies; however, it is not the most promising of our target SE-lncRNAs.
