# Supplementary material for: Cis-acting super-enhancer lncRNAs as biomarkers to early-stage breast cancer
Source: Breast Cancer Res. 2021 Oct 30;23:101. doi: 10.1186/s13058-021-01479-8 (PMC8557595; doi:10.1186/s13058-021-01479-8)
Supplement: Supplementary file 3 — Additional file 3: Figure S3: Specificity of H3K27ac ChIP antibody. Fold enrichment of the H3K27ac antibody between control promoter region of Myt1 and enhancer region of MYC. [file 13058_2021_1479_MOESM3_ESM.docx]

**
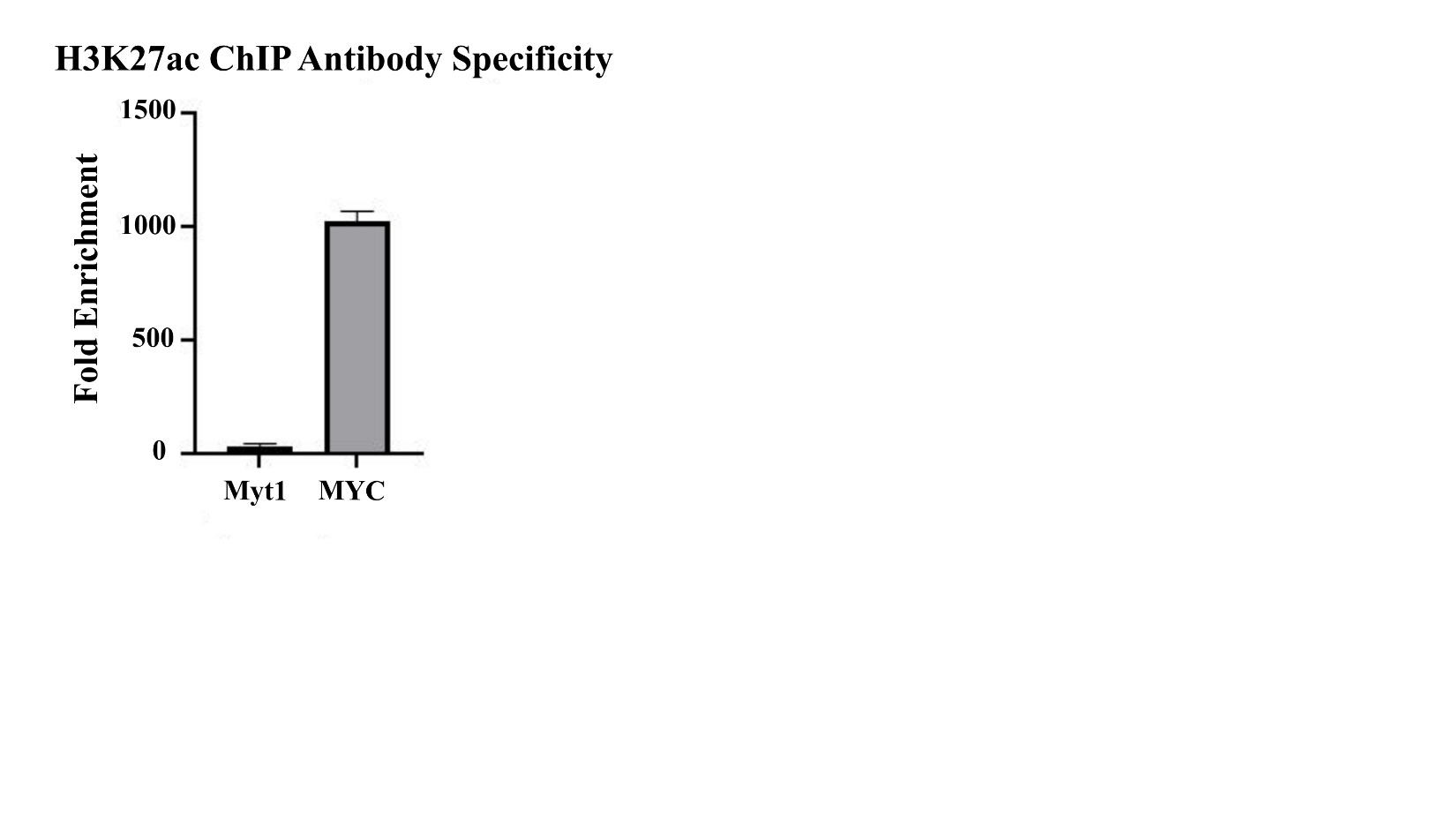
**

**Supplementary Figure 3: Specificity of H3K27ac ChIP Antibody**. Fold enrichment of the H3K27ac antibody between control promoter region of Myt1 and enhancer region of MYC.
